# Introduction of alkali-labile units into lignin in transgenic plants by genetic engineering

**DOI:** 10.1186/1753-6561-5-S7-O56

**Published:** 2011-09-13

**Authors:** Yasuyuki Ishikawa, Yukiko Tsuji, Kanna Sato, Amiu Shino, Yoshihiro Katayama, Jun Kikuchi, Hirofumi Hara, Shojiro Hishiyama, Eiji Masai, Shinya Kajita

**Affiliations:** 1 Graduate School of Bio-Applications and Systems Engineering, Tokyo University of Agriculture and Technology, Koganei, Tokyo 184-8588, Japan; 2 Metabolomics Research Group, RIKEN Plant Science Center, Yokohama, Kanagawa 230-0045, Japan; 3 Department of Forest Science and Resource, College of Bioresource Sciences, Nihon University, Fujisawa, Kanagawa 252-8510, Japan; 4 Department of Biomedical Engineering, Okayama University of Science, Okayama, Okayama 700-0005, Japan; 5 Department of Biomass Chemistry, Forestry and Forest Products Research Institute, Tsukuba, Ibaraki 305-8687, Japan; 6 Department of Bioengineering, Nagaoka University of Technology, Nagaoka, Niigata 940-2188, Japan

## Background

**Figure 1:**
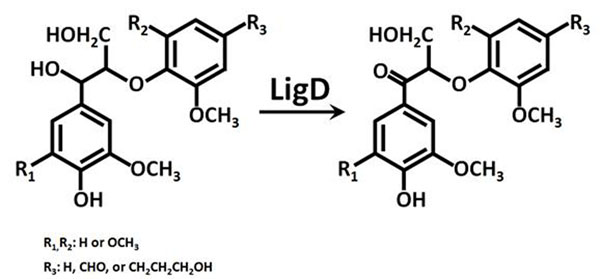
The reaction catalyzed by LigD. This bacterial enzyme catalyzes oxidation of benzyl position of 8-O-4' lignin dimers. Both of guaiacyl-guaiacyl and syringyl-syringyl dimers can be oxidized by the enzyme.

Lignin is one of major components of plant secondary cell wall. In plant cell wall, it is synthesized via radical coupling of precursors such as *p*-coumaryl, coniferyl, and sinapyl alcohols. In early stage of the lignification, 8-O-4’, 8-8’ and 8-5’ dimers are thought to be synthesized mainly from the precursors in the wall. A gram-negative bacterium, *Shingobium* sp. strain SYK-6 (hereafter refer to as SYK-6) is able to catabolize a wide variety of phenolic compounds including the lignin precursors by its unique enzymatic system. One of catabolic enzymes, LigD, catalyzes oxidation at alpha (benzyl) position of 8-O-4’ dimers and forms carbonyl group at the position (Figure 1). This oxidation is the first step of catabolic pathway of 8-O-4’ dimers in SYK-6. When we express LigD polypeptide in the cell wall of transgenic plants, the oxidative dimers will be expected to be generated and then incorporated into lignin polymer. In some past studies, it has been shown that the presence of carbonyl groups at the alpha position of aryl propane units in lignin greatly speeds up the rate of cleavage of beta-aryl ether linkages during kraft pulping condition [1,2]. In order to contribute to efficient and sustainable production of kraft pulp and the other biomass-derived products such as bioethanol, we introduced the *ligD* gene into *Arabidopsis* and hybrid aspen and tried to generate transgenic plants whose lignin can be easy to remove from hollocellulose fraction under alkaline conditions.

## Method

Because of codon usage is significantly different between genes in plants and SYK-6, we chemically synthesized open reading frame (ORF) of the *ligD* gene for improving its expression in the transgenic plants. After addition of nucleotide sequence for apoplast-targeting signal peptide to the synthesized *ligD* ORF, it was introduced into *Arabidopsis thaliana*, tobacco BY-2 and hybrid aspen under the control of cauliflower mosaic virus 35S promoter. LigD expression in the transgenic plants was monitored by Western blot analysis and enzymatic activity with crude extract prepared from each transgenic line. Preliminary analysis of lignin structure by 2D-NMR and nitrobenzene oxidation was also performed.

## Results and discussion

At first we confirmed expression of the *ligD* transgene in *Arabidopsis* by Western blot analysis with antiserum against LigD polypeptide. Positive expressions of the *ligD* were detected in some of the transgenic plants analyzed. Enzymatic activities of LigD in crude extracts prepared from both cytosolic and apoplastic fractions of the transgenic *Arabidopsis* plants were also detected, but it was relatively higher in the latter case. As expected, 2D NMR (^1^H-^13^C HMQC) analysis suggests that the abundance of the alpha-keto (alpha carbonyl) structure in 8-O-4’ units of lignin in the transgenic plants is relatively higher than that in the wild-type plant. Chemical compositions of lignin (syringyl/guaiacyl ratio) and neutral sugars could not be distinguishable between the transgenic and wild-type plants. Generation of transgenic hybrid aspen and its analysis are now in progress.
